# Perceived competence and attitudes towards patients with suicidal behaviour: a survey of general practitioners, psychiatrists and internists

**DOI:** 10.1186/1472-6963-14-208

**Published:** 2014-05-08

**Authors:** Tine K Grimholt, Ole R Haavet, Dag Jacobsen, Leiv Sandvik, Oivind Ekeberg

**Affiliations:** 1 Department of Acute Medicine, Oslo University Hospital, Pb. 4950 Nydalen, Oslo 0424, Norway; 2 Regional Centre of Violence, Traumatic Stress and Suicide Prevention Eastern Norway, Oslo, Norway; 3 Department of General Practice, Institute of Health and Society, University of Oslo, Oslo, Norway; 4 Department of Biostatistics Oslo University Hospital, Oslo, Norway; 5 Department of Behavioural Sciences in Medicine, Institute of Basic Medical Sciences, Faculty of Medicine, University of Oslo, Oslo, Norway

**Keywords:** Attitudes, Incurable illness, Substance misuse, Suicide attempt, Physicians

## Abstract

**Background:**

Competence and attitudes to suicidal behaviour among physicians are important to provide high-quality care for a large patient group. The aim was to study different physicians’ attitudes towards suicidal behaviour and their perceived competence to care for suicidal patients.

**Methods:**

A random selection (n = 750) of all registered General Practitioners, Psychiatrists and Internists in Norway received a questionnaire. The response rate was 40%. The Understanding of Suicidal Patients Scale (USP; scores < 23 = positive attitude) and items about suicide in case of incurable illness from the Attitudes Towards Suicide Questionnaire were used. Five-point Likert scales were used to measure self-perceived competence, level of commitment, empathy and irritation felt towards patients with somatic and psychiatric diagnoses. Questions about training were included.

**Results:**

The physicians held positive attitudes towards suicide attempters (USP = 20.3, 95% CI: 19.6–20.9). Internists and males were significantly less positive. There were no significant differences in the physicians in their attitudes toward suicide in case of incurable illness according to specialty. The physicians were most irritated and less committed to substance misuse patients. Self perceived competence was relatively high. Forty-three percent had participated in courses about suicide assessment and treatment.

**Conclusions:**

The physicians reported positive attitudes and relatively high competence. They were least committed to treat patients with substance misuse. None of the professional groups thought that patients with incurable illness should be given help to commit suicide.

Further customized education with focus on substance misuse might be useful.

## Background

Competence and attitudes influence clinical practice as well as patients experience and outcome [1]. Negative attitudes towards suicide attempters may reduce the ability to address the problems patients are struggling with [2]. Rejection may therefore contribute to increased suicide risk [3].

A review of patients’ experiences after a suicide attempt described poor communication and a lack of staff knowledge [4]. This review described the nature of care reported by service users in a way that indicated that improvements to the attitudes in health settings were still needed to ensure a high-quality service. The users reported that focus was only given to their physical condition and not their mental problems.

Physicians have different attitudes to various diseases, and mental disorders generally have lower status [5]. Lack of sympathy and irritation to self harm were first documented more than 30 years ago [6]. In 2011 results from a review by Saunders and colleagues concluded that attitudes general hospital staff were largely negative [1]. They also found that self harm patients were viewed more negatively than other patients apart from those abusing alcohol and drugs. Differences have been found between health-care professionals’ perceptions of suicide. When psychiatrists were compared with other physician specialists, they showed a more positive attitude and a greater willingness to help [7]. More positive attitudes towards suicide attempters among females have also been demonstrated [8-10]. Physicians in general practice (GP), psychiatry and internal medicine treat suicidal patients in different health care settings. Further, they meet patients at all stages in the suicidal process, ranging from suicidal thoughts to suicide attempts and even completed suicide. Internists will often meet patients in an acute crisis just after a suicide attempt, and often for a short time until the patients are treated somatically. Several patients with somatic illness get depressed, and some even suicidal. Physicians in psychiatric wards and outpatient clinics more often treat the most severely suicidal patients, often referred from GPs and internists and for longer periods. The latter group has also chosen to work within the field of psychiatry and more often have specific training in assessment and treatment.

In case of incurable illness and overwhelming suffering, requests for and performance of euthanasia do not seem to be a rare occurrence [11,12]. In European countries a minority of physicians declared themselves willing to assist a patient when he/she intends to commit suicide, but willingness varied from 12 up to 43%. Different attitudes have also been found in relation to the physicians’ specialty [13].

The present study was designed to determine whether differences exist between GPs and physicians in internal medicine and psychiatry regarding:

1) Attitudes towards patients with suicide attempt in general and specifically towards suicide in case of incurable illness.

2) Whether these attitudes are related to gender, age, clinical experience with suicide and theoretical training in suicidology.

3) Self-perceived competence, commitment, empathy and irritation towards patients with: Somatic diagnoses, substance misuse, depression and anxiety and suicidal behaviour.

4) Interest, perceived skills and need for education in suicidology.

## Methods

### Study participants

During spring 2010 we performed a cross-sectional survey from a random selection of all registered physicians in general practice, psychiatric and medical departments in Norway. The physicians are registered with their work address and email address in a database provided by the Norwegian company Cegedim. The total sample in this study consisted of 750 physicians. An equal sized sample (n = 250) from each group was invited. All participants received a letter of informed consent in accordance with regulations from the Personal Privacy Protection Agency in Oslo University Hospital and a questionnaire by post, as well as an email containing a link to an electronic version of the questionnaire.

### Measures

#### Understanding of suicidal patients scale

We used the Understanding of Suicidal Patients Scale (USP) [8] to measure physicians’ attitudes to suicide attempters. The scale consists of 11 items scored on a five-point scale from 1 (I agree completely) to 5 (I disagree completely). These items are the last eleven in the questionnaire. The scale indicates willingness to provide care for, and understanding and sympathy of, suicide attempters. The total score range is from 11 (completely positive) to 55 (completely negative). Some items are reversed. In a previous study from Finland, a USP score lower than 23 was defined as a positive attitude, whilst a score higher than 33 defined a negative attitude to suicide attempters [14]. The reliability measure of the USP scale in the original study was a Cronbachs alpha of 0.74 [8].

#### Incurable illness

We added three questions from the Attitudes Towards Suicide Questionnaire [15] about physicians’ views of suicide among people with incurable illness: (1) “Suicide should be accepted as a way to shorten an incurable illness”, (2) “I understand that people who suffer from a serious incurable illness may want to end their life”, and (3) “A person suffering from a serious, incurable disease, who specifically asks for help to die, should get that help” Responses were scored on a scale from 1 (I agree completely) to 5 (I disagree completely).

#### Education and experience with suicide

We added a question: “Have you participated in courses or training in the assessment and treatment of patients with suicidal behaviour during the last five years?” Response options were “yes” and “no”. We also used two items about perceived skills and the need for further training [8]: (1) “I think my present competence provides me with skills to care for suicide attempters” and (2) “I am in need of further training to be able to work with people who have tried to end their life”. Responses were scored on a scale from 1 (I agree completely) to 5 (I disagree completely). (The third item in the questionnaire is not used in this paper) To investigate whether experience with patients suicide in own practice was related to the attitudes, we added the question: “Have you ever experienced that one of your patients have committed suicide?” No or yes? And if yes they were asked to provide the number of experienced suicides in an open box.

#### Other comparisons

To compare physicians’ competence, commitment, empathy and irritation toward suicide attempters, and patients with psychiatric, substance misuse and somatic diagnoses we randomly listed the following disorders: Infectious diseases, diabetes, suicidal behaviour, heart disease, cancer, anxiety, depression, misuse of Class A-tranquilizers (narcotics), misuse of opiates and tranquilizers, and misuse of alcohol. All items were scored on a five-point scale ranging from 1 (very low) to 5 (very high). Infectious diseases, diabetes, heart disease and cancer were combined and presented into the category somatic diagnoses. Misuse of class A-tranquilizers (narcotics), misuse of opiates and tranquilizers, and misuse of alcohol were combined into the category substance misuse. The questionnaire used in the survey can be downloaded here (Additional file 1).

### Statistics

Descriptive statistics, means and frequencies were used. Cronbach’s alpha for the USP-scale was 0.69, indicating acceptable reliability. The chi-square test was used for the comparison of categorical data. Students’ *t*-test and one-way ANOVA were used to compare continuous data between groups. To adjust for the background variables of gender, age, course participation and having experience treating suicidal patients, multivariate linear regression analyses was used. In accordance with the preconditions for the latter analyses, we verified that residuals were sufficiently close to the normal distribution and that colinearity was acceptable for each covariate (VIF <5). A 5% significance level was used. The data was analyzed with SPSS software (v. 18; IBM, Armonk, NY, USA).

### Ethics

The study was approved by the Oslo University Hospital’s Privacy Protection Supervisor, and performed in accordance with the data storage and confidentiality regulations. To maintain confidentiality, we classified age and experience as a physician into categories rather than using exact values. Personal information was kept separate from the questionnaires and could only be accessed and identified by a series of numbers from a code list.

## Results

### Sample characteristics

After three written reminders, 300 (40%) physicians answered the questionnaire by mail. None of the physicians used the link they had received via email to respond to the electronic form of the questionnaire.

As shown in Table 1, the response rate did not differ significantly according to the physicians’ specialty. There were significantly more females among the psychiatrists. The physicians in internal medicine were significantly younger. A significantly larger proportion of GPs and psychiatrists had experienced suicide in a patient of their own compared with internists.

**Table 1:** Sample characteristics of the participants, specialty, gender, experience, age and experience of suicide in patients (n = 300)

	**General practice**	**Psychiatry**	**Internal medicine**	**p-value**^**a**^
N = 300/750	250	250	250	
	n (%)	n (%)	n (%)	
	91 (36)	102 (41)	107 (43)	
**Female gender**	31 (34)	48 (47)	30 (28)	0.015
**Experience (years)**				
0–5	4 (4)	7 (7)	29 (27)	<0.001
6–10	11 (12)	14 (14)	16 (15)	
11–20	25 (28)	29 (28)	20 (19)	
21–30	30 (33)	25 (25)	21 (20)	
>30	19 (21)	27 (27)	21 (20)	
Missing	2 (2)			
**Age groups (years)**				<0.001
< 30	–	–	12 (11)	
30–40	16 (18)	26 (26)	37 (35)	
41–50	28 (31)	26 (26)	22 (21)	
51–60	35 (39)	25 (25)	24 (22)	
>60	12 (13)	24 (24)	12 (11)	
Missing	-	1 (1)	-	
**Experienced suicide**	68 (75)	73 (72)	38 (36)	<0.001

^a^Chi-square test and ANOVA.

### Attitudes

#### Understanding of and willingness to provide care for suicide attempters

The mean USP score was 20.3 (95% CI: 19.6–20.9). As shown in Table 2, the mean USP scores were all below 23, indicating a positive attitude towards suicide attempters among all three groups of physicians. There were significant differences between the three groups; psychiatrists had the lowest mean score followed by GPs and internists. The *t*-tests between genders in each group showed that the only significant difference between females and males was in general practice (Table 2).

**Table 2:** Attitudes measured with the Understanding of Suicidal Patients Scale according to gender and specialty

	**General practitioners**	**Psychiatrists**	**Internists**
	**(n = 85)**	**(n = 97)**	**(n = 105)**
	**Mean (95% CI)**	**Mean (95% CI)**	**Mean (95% CI)**
Total	19.4 (18.3–20.4)	18.4 (17.5–19.4)	22.7 (21.7–23.7) p = 0.01
Females	17.7 (16.2–19.2)	18.3 (16.7–19.9)	21.7 (20.0–23.4)
Males	20.3 (19.0–21.6)	18.3 (17.4–19.7)	23.0 (21.8–24.3)
	p = 0.014	p = 0.785	p = 0.245

The Understanding of Suicidal Patients Scale ranges from 11 (completely positive) to 55 (completely negative). Scores below 23 are considered to be positive.

The adjusted model also showed significant differences between females and males in total (β = −1.3, 95% CI: −2.6 to −0.06, p = 0.04).

The adjusted multivariate regression model showed significantly more positive attitudes towards suicidal patients among GPs and psychiatrists than among internists (Table 3). There were no significant differences in course participation or age in the adjusted model.

**Table 3:** Multivariable comparisons of attitudes between general practitioners, psychiatrists and internists with the Understanding of Suicidal Patients Scale as dependent variable

	**Unadjusted**			Adjusted*		
	**β**	**95% CI**	**p-value**	**β**	**95% CI**	**p-value**
Psychiatrists vs. General Practitioners	−0.95	−0.4 to 2.3	0.18	−0.6	−2.1 to −0.8	0.393
General Practitioners vs. Internists	−3.3	−4.8 to −1.8	<0.001	−2.3	−4.0 to – 0.6	0.009
Psychiatrists vs. Internists	−4.3	−5.6 to −2.9	<0.001	−3.9	−5.8 to −1.9	<0.001

*Adjusted for age, gender, course participation and experience of suicide among patients.

Items from the USP showed that overall all three groups of physicians understood that suicidal patients have emotional problems and therefore need the best possible treatment (1.4, 95% CI: 1.4–1.5). They also agreed with the item: “A person who has tried to commit suicide is a person I would like to help” (1.5, 95% CI: 1.4–1.6). Psychiatrists (1.7, 95% CI: 1.6–1.9) responded significantly more often than GPs (2.0, 95% CI: 1.8–2.2) and internists (2.3, 95% CI: 2.1–2.5) that patients were treated well in their unit (p < 0.001). The internists were less likely to agree with the statement “I try to do my best to talk with a patient who has attempted suicide about his or her personal problems” (2.0, 95% CI: 1.8–2.2) than GPs (1.5, 95% CI: 1.3–1.6) and psychiatrists (1.4, 95% CI: 1.2-1.6) (p < 0.001). Internists were more likely to agree with the statement “I often find it difficult to understand a person who has tried to commit suicide” (3.4, 95% CI: 3.2–3.7) than GPs (2.8, 95% CI: 2.6–3.1) and psychiatrists (2.4, 95% CI: 2.2–2.6). Likewise, internists were more likely to agree with the statement “It is usually troublesome to treat a patient who has tried to commit suicide” (3.1, 95% CI: 2.9–3.4) than GPs (2.5, 95% CI: 2.2–2.8) and psychiatrists (2.4, 95% CI: 2.1–2.7) (p < 0.001).

### Suicide among patients with incurable illness

Responses to the three questions about incurable illness showed no significant differences between the physicians’ specialties. For the item “Suicide should be accepted as a way to shorten an incurable illness”, the mean score was almost neutral (3.6, 95% CI: 3.5–3.8). For the item “I understand that people who suffer from a serious incurable illness may want to end their life”, the mean score was 2.4 (95% CI: 2.3–2.5), indicating slight agreement. A mean score of 4.1 (95% CI: 3.9–4.2) for the statement: “A person suffering from a serious, incurable disease, and specifically asks for help to die, should get that help” indicated that all groups disagreed.

Females (3.9, 95% CI: 3.6–4.1) were significantly less likely than males (3.5, 95% CI: 3.3–3.7) to agree with the statement that suicide was an acceptable way to shorten an incurable illness (p = 0.028). Although not statistically significant, females were less understanding of suicide among people with incurable illnesses (2.6, 95% CI: 2.3–2.8) than males (2.3, 95% CI: 2.1–2.5) (p = 0.06). There was no difference between the responses of females (4.2, 95% CI: 4.0–4.4) and males (4.0, 95% CI: 3.8–4.2) on the statement that a person with incurable illness should be able to get help to end his/her life (p = 0.279).

### Self-perceived competence, commitment, empathy and irritation in regard to psychiatric and somatic diagnoses and substance misuse

#### Self-perceived competence

The levels of perceived competence were generally aligned with the physicians’ specialty (Table 4). Psychiatrists perceived their competence to be highest to treat patients with psychiatric and substance misuse diagnoses. In contrast the internists perceived their competence to be highest for the somatic diagnoses. The GPs competence scores were between above the middle values (3.2 and 3.7) for all diagnoses. All physicians reported higher levels of competence in treating depression and anxiety than suicidal behaviour. Similar results were obtained when adjusting for age and gender.

**Table 4:** Self-perceived competence, commitment, empathy and irritation with somatic diagnoses, substance misuse, depression and anxiety and suicidal behaviour according to specialty

	**General practitioners mean (95% CI)**	**Psychiatrists mean (95% CI)**	**Internists mean (95% CI)**	**p-value**
**Competence**				
Scale range: 1 (very low) to 5 (very high)				
Somatic diagnoses	3.6 (3.5–3.7)	2.3 (2.1–2.4)	3.6 3.5–3.7)	<0.001
Substance misuse	3.3 (3.1–3.4)	3.5 (3.4–3.6)	2.5 (2.4–2.6)	<0.001
Depression and anxiety	3.6 (3.4–3.7)	4.3 (4.2–4.4)	2.5 (2.3–2.6)	<0.001
Suicidal behaviour	3.2 (3.1–3.4)	4.1 (4.0–4.2)	2.4 (2.3–2.5)	<0.001
**Commitment**				
Scale range: 1 (very low) to 5 (very high)				
Somatic diagnoses	3.7 (3.6–3.8)	2.3 (2.2–2.5)	3.7 (3.6–3.8)	<0.001
Substance misuse	3.4 (3.3–3.5)	3.8 (3.7–3.9)	2.6 (2.5–2.8)	<0.001
Depression and anxiety	3.7 (3.6–3.8)	4.2 (4.1–4.3)	2.7 (2.5–2.8)	<0.001
Suicidal behaviour	3.7 (3.5–3.8)	4.2 (4.1–4.3)	2.8 (2.6–3.0)	<0.001
**Empathy**				
Scale range: 1 (low) to 5 (very high)				
Somatic diagnoses	3.6 (3.5–3.8)	3.4 (3.3–3.5)	3.8 (3.7–3.9)	<0.001
Substance misuse	3.0 (2.9–3.2)	3.2 (3.0–3.3)	2.9 (2.8–3.1)	0.122
Depression and anxiety	3.8 (3.7–3.9)	3.9 (3.8–4.1)	3.5 (3.4–3.7)	<0.001
Suicidal behaviour	3.9 (3.8–4.1)	3.9 (3.8–4.1)	3.7 (3.6–3.9)	0.076
**Irritation**				
Scale range: 1 (not at all irritated) to 5 (very irritated)				
Somatic diagnoses	1.2 (1.1–1.3)	1.2 (1.1–1.2)	1.2 (1.1–1.3)	0.668
Substance misuse	2.6 (2.4–2.8)	2.4 (2.2–2.6)	2.5 (2.3–2.7)	0.325
Depression and anxiety	1.6 (1.4–1.7)	1.5 (1.4–1.6)	1.5 (1.4–1.6)	0.776
Suicidal behaviour	1.6 (1.4–1.8)	1.9 (1.7–2.1)	1.8 (1.6–1.9)	0.148

Mean values and 95% CI from one-way ANOVA.

#### Commitment

There were significant differences between internists and psychiatrists with regard to which diagnoses they were most committed to (Table 4), with almost the same pattern as shown for self-perceived competence. The commitment was lower, with scores ranging from slightly low empathy to neutral empathy among all three groups of physicians for misuse of Class B-tranquilizers (2.5–3.5), Class A-tranquilizers and opiates (2.5–3.2) and alcohol (2.6–3.4) compared with the psychiatric diagnoses of depression and anxiety (2.7–4.2) and suicidal behaviour (2.8–4.2).

#### Empathy

There were no differences between the physicians’ specialties in empathy to suicidal behaviour and substance misuse. Females were significantly more empathetic to suicide attempters than males (4.0 vs. 3.8), even when responses were adjusted for profession, age and course participation) (p = 0.002). Depression and cancer were the diagnoses that received the highest levels of empathy with mean scores from 3.6 - 4.0 and 3.9 - 4.2, respectively. Patients with substance misuse received the lowest empathy scores from all three groups (2.9–3.2).

#### Irritation

Compared with the other diagnoses, the substance misuse category received highest irritation scores (2.4–2.6) (Table 4). The physicians were not irritated by patients with suicidal behaviour (1.6–1.9). There were no significant differences according to specialty or gender.

#### Interest, skills and need for education

Forty-three percent (n = 128) of the physicians in our sample had participated in courses or other forms of education about the assessment and treatment of suicidal patients during the last five years. Psychiatrists had a significantly higher participation rate (n = 77, 76%) than GPs (n = 35, 39%) and internists (n = 16, 15%) (p < 0.001). The psychiatrists were most interested in courses and training (2.5, 95% CI: 2.3–2.7) whilst the internists were less interested (3.4, 95% CI: 3.2–3.5) (p < 0.001).

The internists perceived that their previous training had given them intermediate good skills (3.2, 95% CI: 3.0–3.4) in taking care of people who had tried to commit suicide. The scores among the GPs were (2.4, 95% CI: 2.2–2.6) and among the psychiatrists (1.7, 95% CI: 1.5–1.9) (p < 0.001). The internists were more likely to agree that they needed further training (3.8, 95% CI: 3.6–4.1) than GPs (3.8, 95% CI: 3.5–4.0) and psychiatrists (3.1, 95% CI: 2.9–3.4) (p < 0.001).

## Discussion

In the present study we found that, patients with misuse problems received lowest commitment, empathy and generated more irritation than patients with other diagnoses. These findings have not to our knowledge been presented before. The psychiatrists and GPs had somewhat more positive attitudes to suicidal patients than the internists, and female physicians were more positive than males. This study shows that all three groups of physicians had positive attitudes towards suicidal patients and were understanding and willing to help them. The levels of competence and commitment tended to align with the physicians’ areas of specialization. The finding that the physicians were most irritated by and least committed to treat substance misuse patients than patients with other diagnoses is concerning. Substance misuse patients often have large and complex problems, and increased morbidity and mortality rates [16]. In the review of Saunders et al. [1], attitudes among health care professionals were largely negative. Compared to these findings, however,the present study show that physicians report more positive attitudes,and willingness to help suicidal patients. The results are consistent with another study where more positive attitudes have been found among health-care professionals working in psychiatric wards than those working in other fields [8]. This is also in line with a study of professionals in mental health-care in Norwegian outpatient clinics [17].

### Incurable illness

In our study, physicians showed a higher degree of understanding than acceptance of suicide among people with an incurable illness. In Norway, as in many other countries, euthanasia is illegal. The distinction between suicide among patients with incurable illnesses and other serious somatic and psychiatric diagnoses is delicate. It is important that this issue is addressed because it raises ethical questions and should be considered when educational initiatives are raised. During a medical career, there is a probability that a patient may, by implication or directly, ask for help to end his or her life. It is therefore important that physicians have reflected on their own personal and professional attitudes. Even though all groups stated that patients who ask for help to commit suicide should not receive it, they were able to understand why patients with an incurable disease may want to end their life. This reflects an understanding attitude for patients’ decisions, but maintains the premise that the health-care professional’s role is not to help patients end their lives.

### Competence

The levels of competence and commitment tended to correspond with the physicians’ areas of specialization. Training can improve clinicians’ attitudes toward suicide, confidence in working with clients at risk of suicide, and, most importantly, their skills in clinical practice [18]. A survey in London showed that 25% of all people who completed suicide had seen their GP during the previous month [19]. Consistent with the findings of our study, GPs are interested in further training [20]. It has been shown that directed efforts of GPs can reduce the number of suicides [21]. In study by Poma et al., sixty-one percent of doctors admitted difficulties in exploring suicidal ideation, but tended to ascribe this to a reluctant attitude of patients [22]. The study underscores the finding that GPs’ need assistance in the difficult task of recognizing suicidal patients. Apparently, there is a lack of instruments available for GPS to discover suicidal risk at an early stage [23].

Fifteen percent of the internists had participated in courses in assessment and treatment of suicidal patients during the last five years. This is probably somewhat less than optimal. However, it should be emphasized that the need for such training is most important to the staff in accident and emergency departments, because they deal with suicide attempt patients more frequently. In a review of self-harming patients’ attitudes to clinical services, the main points for improved patient satisfaction were increased knowledge and communication skills among staff [4]. In our study, the internists found it more difficult than GPs and psychiatrists to talk to patients after a suicide attempt. All three professional groups, however, frequently encounter patients with depression, substance misuse and personality disorders, and should have the competence to detect suicidal risk.

### Methodological considerations

The generalizability of the findings is limited by the low response rate. However, the relative differences between the groups’ specialties and gender are probably still reliable because of the robust sample size and consistent figures. We used a previously validated scale to measure health-care professionals’ attitudes and the reliability of the scale was found satisfactory.

A review study of scales used to measure attitudes to suicidal behaviour [24] concluded that there are other, more reliable, scales than the USP. The USP was developed specifically to measure health-care professionals’ attitudes to patients after a suicide attempt, and was therefore found to be most suitable for the specific research questions in our study.

In a study by Creed and Pfeffer, the results showed more positive attitudes among physicians towards somatic diagnoses than self harm [25]. There is no available validated scale for this purpose, so in the present study a new self rating scale was used in order to determine whether this was still present and compare the current attitudes to somatic diagnoses and substance misuse with these findings. The disorders were selected because they are common, and all clinical physicians have treated these patients.

Because the subject of this study is sensitive, a completely anonymous survey may have increased the response rate. To increase the level of discretion and decrease concern among the participants that stating their exact age and information of their profession might make them recognizable, we therefore used categories of age and experience rather than the exact values. The use of electronic questionnaires did not facilitate participation; no participants chose to answer in this way. It should also be considered that those responding might be more dedicated and interested in the current topic and therefore represent a response bias. Unfortunately we did not have the opportunity to perform drop out analyses in this study.

### Clinical interpretations and relevance

The relevance of attitudes in clinical practice has previously been emphasized, because attitudes may influence care decisions. If the commitment in clinical practice is consistent with the findings in this study, it appears that patients with substance misuse problems receive the least attention.

We know that working with suicidal patients can be stressful and demanding, especially because of the continuous threat that these patients may eventually take their own lives. Colson et al. [26] underlined the importance of staff members’ perceptions that some patient groups are difficult to treat, and suggested that this could influence the patients and the treatment process, with implications for progress and prognosis. They found that patients with suicidal depressed behaviour were one of the groups most commonly associated with staff members’ perceptions of being difficult to treat. They suggest that continuous supervision is important to prevent negative attitudes and anxiety of failure, especially among physicians with responsibility for treatment.

To prevent these factors, and to increase confidence in treating patients in a suicidal crisis, continuously updated knowledge is needed, especially with regard to assessment of suicidal risk and treatment options. This is highlighted in the National Institutes for Clinical Excellence guidelines on the care of people who self-harm [27]. The findings in this study underline the importance of implementing appropriate training in basic medical education, regular internal training and continued education. Such educational initiatives should probably be modified for each group of physicians, so they are perceived as relevant and meaningful to their specific situation. Half of the participants reported participation in some form of suicidology education during the previous five years. The next step is to determine how such education can be made more readily available to as many physicians as possible. A successful example was the STORM project, in which training of professionals in primary and mental health care and accident and emergency departments showed improved skills in the assessment and management of suicide risk, and high levels of satisfaction with the training [28].

### Future research

Because the results might be influenced by social desirability, an indirect way of measuring general attitudes in the wards could have been to ask the physicians what they thought of their colleagues’ attitudes. This technique was used by Album and Westin, [5] who asked physicians how they thought other physicians would prioritize different diagnoses, and found that patients with depression and anxiety received the lowest scores. Further, as Taylor et al. suggest, [4] more insight could be obtained from patients’ own experiences with different parts of the health-care system using standardized interview schedules. Finally, to make educational initiatives more interesting, further research should also investigate what kind of training and education professionals might find useful in their clinical work.

## Conclusions

The attitudes toward treating people with suicidal behaviour were generally positive, particularly among GPs, psychiatrists and female physicians. Physicians were least committed to treat substance misuse patients compared with patients with other diagnoses. None of the professional groups thought that patients with incurable illness should be given help to commit suicide. The levels of perceived competence and commitment reflect the physicians’ areas of specialization. All groups reported a moderate interest in more training. In the future, educational programs aimed at each specialty and their current challenges and needs might be useful.

## Competing interest

The authors declare that they have no competing interests.

## Authors’ contributions

TKG designed the study, organized the collection of data, analyzed the data and drafted the manuscript. ORH and DJ critically revised and contributed to the manuscript. LS gave statistical advice and controlled the analyzes. OE designed the study, supervised the work, analyzed data and revised the manuscript. All authors have read and approved the latest version of the manuscript before it was admitted.

## Pre-publication history

The pre-publication history for this paper can be accessed here:

http://www.biomedcentral.com/1472-6963/14/208/prepub

## Supplementary Material

Additional file 1 The full version of the questionnaire used in the survey. Click here for file
