# Supplementary material for: Perceived competence and attitudes towards patients with suicidal behaviour: a survey of general practitioners, psychiatrists and internists
Source: BMC Health Serv Res. 2014 May 8;14:208. doi: 10.1186/1472-6963-14-208 (PMC4048050; doi:10.1186/1472-6963-14-208)
Supplement: Additional file 1 — The full version of the questionnaire used in the survey. [file 1472-6963-14-208-S1.doc]

**1. Gender:** Male  Female

**2. Age group**

30 or less 31-40 41-50 51-60 More than 60 yrs

**3. Are you an authorized specialist? Yes No**

General Practice

Psychiatry

Internal Medicine

**4. How many years have you practiced as a physichian?**

0-5 yrs

6-10 yrs

11- 20 yrs

21-30 yrs

More than 30 yrs

5. Have you participated in courses or training in assessement and treatment of patients with suicidal behaviour during the last five years?

Yes  if yes Course

No  Other training:___________________________

# 6. If yes, approximately how many hours have you participated in course or other training in assessement and treatment of patients with suicidal behaviour during the last five years?

Courses Other training

0 hours

1-9 hours

10-20 hours

> 20 hours

> 30 hours

**7. To what degree are you interested in courses and training in suicidology?**

Not at all To a little To some To a rather high To a very

degree degree degree high degree

**8. Have you ever experienced that one of your patients have committed suicide?**

No  Yes  Numbers_____

# 9. How do you rate your competence to treat patients with various disorders?

1= Very low competence, 3= Intermediate competence, 5 = Very high competence

Very low Intermediate Very high

competence competence

**1 2 3 4 5**

Infectious diseases

Diabetes mellitus

Suicidal behaviour

Heart diseases

Anxiety disorders

Misuse of b-tranquilizers

Misuse of alcohol

Depression

Misuse of a- tranquilizers and

opiates

Cancer

10. Your personal commitment to patient groups with various disorders?

1= Very low degree of commitment, 3= Intermediate, 5 = Very high degree of commitment

Very low Intermediate Very high degree

Degree of of commitment

commitment

**1 2 3 4 5**

Infectious diseases

Diabetes mellitus

Suicidal behaviour

Heart diseases

Anxiety disorders

Misuse of b-tranquilizers

Misuse of alcohol

Depression

Misuse of a- tranquilizers and

opiates

Cancer

1. How do you rate your level of empathy in relation to patients with various disorders?

1= Low degree of empathy, 3= Intermediate, 5 = Very high degree of empathy

Low degree Intermediate Very high degree

of empathy of empathy

**1 2 3 4 5**

Infectious diseases

Diabetes mellitus

Suicidal behaviour

Heart diseases

Anxiety disorders

Misuse of b-tranquilizers

Misuse of alcohol

Depression

Misuse of a- tranquilizers and

opiates

Cancer

12. Your sense of irritation to patients with various diseases

1= Not irritated at all 3= Intermediate 5= Very irritated

**Not irritated Intermediate Very**

**at irritated**

**all**

Infectious diseases

Diabetes mellitus

Suicidal behaviour

Heart diseases

Anxiety disorders

Misuse of b-tranquilizers

Misuse of alcohol

Depression

Misuse of a- tranquilizers and

opiates

Cancer

13. In the following we want to determine your view of: **suicide, suicide attempt and own need for training.** (*Understanding of Suicidal Patients Scale*)

**I agree Neither I disagree**

**Completely agree completely**

**nor**

**disagree**

**1 2 3 4 5**

I think my present training has

provided me with adequate skills

to take care of people who have tried to

commit suicide

I am in need of further training to be

able to work with people who

have tried to end their life

Mental health services

work well for people who have

tried to commit suicide

Patients who have tried to commit suicide

are usually treated well in my work unit

When I treat a person who have

tried to commit suicide,

I sometimes show my irritation

I am usually sympathetic

and understanding towards

a patient who has tried to commit suicide

I try to do my best to make

a patient who has tried to

commit suicide feel comfortable and secure

It is usually troublesome

to meet a patient who has tried

to commit suicide

I try to do my best to talk with

a patient who has attempted suicide

about his or her personal problems

A person who has made

several suicide attempts

is at great risk of committing suicide

I would like to help a person

who has tried to commit suicide

Because suicidal patients

have emotional problems,

they need the best possible treatment

I treat patients who have tried

to commit suicide

as willingly and sympathetically as other patients

I often find it difficult to understand

a person who have tried to commit suicide

14. Finally, your view of suicide in patients in case of incurable disorder

**I agree Neither I disagree**

**Completely agree completely**

**nor**

**disagree**

Suicide should be accepted as a way

to shorten an incurable illness

I understand that people who suffer

from a serious incurable illness

end their life

A person suffering from a serious,

incurable disease,

and specifically asks for help to die,

should get that help

Thank you very much.
